# Long-term association of remnant cholesterol with all-cause and cardiovascular disease mortality: a nationally representative cohort study

**DOI:** 10.3389/fcvm.2024.1286091

**Published:** 2024-07-08

**Authors:** Min Chen, Zhi Chen, Huarong Ye, Yuanling Cheng, Zhigang Jin, Shaoqian Cai

**Affiliations:** ^1^ Department of Cardiology, China Resources & Wisco General Hospital, Wuhan University of Science and Technology, Wuhan, China; ^2^ Medical College of Wuhan University of Science and Technology, Wuhan, China; ^3^ Department of Ultrasound, China Resources & Wisco General Hospital, Wuhan University of Science and Technology, Wuhan, China

**Keywords:** all-cause mortality, cardiovascular disease, cohort study, general population, remnant cholesterol

## Abstract

**Background and objectives:**

Despite reducing low-density lipoprotein cholesterol (LDL-C) to the normal range, residual cardiovascular risk remain. Remnant cholesterol (RC) exerts a potential residual risk for cardiovascular disease (CVD) prevention, and the long-term longitudinal association between RC and mortality has yet to be well elucidated.

**Methods:**

This study examined a nationally representative sample of 13,383 adults aged 20 years or older (mean age 45.7 and 52% women) who participated in the NHANES III (from1988 to1994). Causes of death were ascertained by linkage to death records through December 31, 2019. The relations of RC with all-cause and CVD mortality were tested using weighted Cox proportional hazard models.

**Results:**

Through a median follow-up of 26.6 years, 5,044 deaths were reported, comprising 1,741 deaths of CVD [1,409 deaths of ischemic heart disease (IHD) and 332 deaths of stroke] and 1,126 of cancer. Compared to those with RC <14.26 mg/dl (lowest quartile), participants with RC ≥29.80 mg/dl (highest quartile) had multivariable-adjusted HRs of 1.23 (95% CI: 1.07–1.42) for all-cause mortality, 1.22 (95% CI: 0.97–1.53) for CVD mortality, and 1.32 (95% CI: 1.03–1.69) for IHD mortality, and 0.89 (95% CI: 0.55–1.43) for stroke mortality, and 1.17 (95% CI 0.90–1.52) for cancer mortality. We observed that elevated RC levels increased CVD risk and IHD mortality despite LDL-C being in the normal range.

**Conclusions:**

Elevated blood RC was associated with an increased long-term risk of all-cause, CVD, and IHD mortality. These associations were independent of socioeconomic factors, lifestyles, and history of diseases, and remained robust across the LDL-C stratum. Measuring RC levels might favor clinical assessment of early CVD risk. Further investigation is needed to elucidate the optimal range of RC levels for cardiovascular disease health in the general population.

## 1 Introduction

Cardiovascular disease (CVD), i.e., ischemic heart disease (IHD) and stroke, is the main cause of global death and a major factor in disability (1). The prevalence of CVD and disease burden are increasing yearly (2). Epidemiological researches demonstrate that CVD accounts for more than 17 million yearly deaths worldwide (3).

It is well known that dyslipidemia, mainly low-density lipoprotein cholesterol (LDL-C), exerts a major risk factor for CVD. Generally, guidelines from many countries recommend controlling LDL-C within normal limits to prevent CVD morbidity and mortality (4). A decrease of 1 mmol/L in LDL-C levels due to statin therapy can lead to a significant 22% reduction in major cardiovascular events over 5 years (5). However, despite LDL-C having dropped to the normal level, there is still a risk for CVD, i.e., a residual cardiovascular risk, which may be attributable to other lipids.

Remnant cholesterol (RC) is a component of triglyceride-rich lipoproteins, very-low-density lipoprotein cholesterol, and intermediate-density lipoprotein cholesterol in the fasting state; in the non-fasting state, it also includes chylomicron (6). Recently, the influence of RC in the development of CVD has raised considerable concern. Varbo et al. suggested that high levels of RC are associated with low-grade inflammation and CVD, whereas LDL-C is only associated with IHD (7). In a Denmark 34-year follow-up prospective study, the risk of IHD increased by increasing 2.8 times for every 1 mmol/L in RC and by increasing 1.5 times for every 1 mmol/L in LDL-C (8). Quispe et al. found that RC adds value in predicting major cardiovascular events independently of traditional risk factors such as LDL-C and apolipoprotein B (9). The studies above provide evidence that elevated RC is related to the occurrence of CVD. However, limited evidence reveals the relationship between RC levels and long-term risk of all-cause and cause-specific mortality. We aimed to evaluate the relationship between the RC levels and risks of all-cause and CVD mortality in a nationally representative cohort with up to 31 years of follow-up in the US general population.

## 2 Methods

### 2.1 Data sources

The experimental data for this study was obtained from NHANES, a publicly available, free, and directly accessible database (https://wwwn.cdc.gov/Nchs/Nhanes).

### 2.2 Study population

The NHANES database is a collection of information on the health and nutritional status of adults and children in the United States, compiled by the Centers for Disease Control and Prevention. The NHANES III dataset was collected from 1988 to 1994, and participants were selected through stratified probability cluster sampling at different stages, representing the health status of all civilians. The interview included demographic, socioeconomic, dietary, and health-related questions. The examination consists of medical, dental, and physiological measurements and laboratory tests administered by trained medical personnel. NHANES was approved by the NCHS Ethics Review Board, and all participants signed an informed consent form all participants.

This study contained 13,383 participants aged 20 years and older (Supplementary Figure S1). In addition, We included participants with available information about High-density lipoprotein cholesterol (HDL-C), total cholesterol (TC), and triglyceride (TG). Participants who were pregnant, had a history of CVD or cancer were excluded from this study. Finally, 13,383 participants were included in our analysis.

### 2.3 Lipid profiles

Enzymatic assays were employed to measure the blood levels of TC and TG, while immunoassays were utilized to assess HDL-C concentrations. LDL-C is calculated using the Friedewald calculation, which involves measuring TC, TG, and HDL-C. The existing studies have shown that the Friedwald equation has good accuracy in LDL-C calculations regardless of whether the subject is fasting or not (10). The level of RC is calculated by subtracting LDL-C and HDL-C from TC (11).

### 2.4 Covariates

Socioeconomic factors, including education level, sex, age, race/ethnicity, and family income, were collected through standard questionnaires in the interview process. Ethnicity was classified into four categories: Mexican American, non-Hispanic Black, non-Hispanic White, and others. Smoking status was graded from never, former, and current smokers. Depending on the daily intake, alcohol can be categorized into: never drinker (0 g/day), moderate drinker (men: <28 g/day and women: <14 g/day), and heavy drinker (men: ≥28 g/day and women: ≥14 g/day). Based on the duration of the activity, physical activity was categorized into inactive group, active group, and underactive group. Participants with no leisure time for physical activity were classified as inactive. Participants who performed strenuous activity at least three times a week were classified as active. In addition, those who actively participated in activities but did not meet the recommended activity standards were defined as the inactive group (12). Educational attainment was divided into four levels: below high school, high school, above high school, and unknown. In this study, family income is determined based on the Median household income t reported in the NHANES III family questionnaire (13). We can then calculate the income-poverty ratio (IPR) based on the ratio of family income to the poverty threshold values. The Census Bureau publishes the national poverty threshold values annually. Generally speaking, family income is directly proportional to IPR, meaning that the higher the IPR, the higher the family income (14). According to the previous report (15), the IPR was categorized as ≤1.30, 1.31–3.50, and >3.50.

We measured overall quality of participants’ diet based on the Healthy Eating Index-2010 (HEI-2010). The score ranged from 0 to 100, with higher scores indicating higher quality (16). The HEI-2010 was calculated by 10 dietary components: grains, fruits, vegetables, dairy products, meats, dietary fats, saturated fats, cholesterol and sodium intake, and variety score. In this study, we take the USDA Automated Multiple-Pass Method to calculate the total energy intake (TEI). A body mass index (BMI) over or equal to 25 was classified as overweight. Diabetes was defined as having a fasting glucose level of 7 mmol/L or higher, glycated hemoglobin A1c level of 6.5% or higher, a history of diabetes, or the use of insulin or oral hypoglycemic agents (17). After the participant remained quiet and rested for five minutes, three consecutive blood pressure readings were taken and averaged as his blood pressure. Hypertension was diagnosed as systolic blood pressure ≥140 mmHg or diastolic blood pressure ≥90 mmHg, a history of hypertension, or taking antihypertensive medications (18).

### 2.5 Outcome ascertainment

Mortality files from the NHANES III dataset as of December 31, 2019, were used for data analysis. The NCHS uses a probability matching algorithm to match this mortality file with the National Death Index (NDI) and calculate the mortality rate (19). The present results indicate that mortality from causes in NDI can effectively classify death categories accurately (20, 21). According to the Ninth revised edition of the International Classification of Diseases (ICD-9) published in 1998, data on potential causes of death are used for case definitions, while the remaining data is used for case definitions based on the tenth revised edition (ICD-10). To adjust for changes between the 2 coding systems, the cause of death before 1999 was recoded into comparable ICD-10-based underlying cause-of-death groups (22). According to ICD-10, the NCHS categorizes mortality into coronary heart disease, stroke (i.e., cerebrovascular disease), and cancer (i.e., malignant tumors). Participants who survived were censored administratively on 31 December 2019. Follow-up time for each participant was calculated from the difference in time between their NHANES III examination date and the last known survival date. In addition, the follow-up time can be calculated by subtracting the NHANES III check date from the date they were removed from the NHANES III mortality file.

### 2.6 Statistical analysis

All data analyses in the NHANES study incorporated a composite, multistage, stratified, and cluster-sampling design. This design included oversampling specific subpopulations. To account for this design, we utilized sample weights, strata, and principal sampling units. In our analyses, we used SDPSTRA6 to represent the stratum, SDPPSU6 to represent the primary sampling units, and WTPFHX6 to represent the final sample weight. Continuous variables were expressed as mean ± standard error, and categorical variables were presented as percentages ± standard error. Cox proportional hazards models were constructed to explore the associations of RC levels with all-cause and cardiovascular mortality. RC levels were categorized according to quartiles: <14.26, 14.26–19.77, 19.77–29.80, and ≥29.8 mg/dl. We took the first quartile of RC as the reference, and hazard ratios (HRs) with 95% confidence intervals (CIs) were calculated. The models were successively adjusted for age, sex, race/ethnicity, education, family income level, smoking status, alcohol intake, physical activity, TEI, HEI-2010, BMI, and history of diabetes and hypertension. To evaluate the model assumptions, we tested the proportional hazards assumption for all analyses. In order to present the non-linear association between RC and mortality, we utilized smooth splines by employing a restricted cubic spline model with four knots using the rms, hmisc, lattice, and survival packages in the R software. For missing values of variables, additional categories are compensated for using dummy coding methods. For subgroup analysis, the results were stratified by sex, age, race/ethnicity, BMI, smoking status, alcohol consumption, physical activity level, diabetes, and hypertension. We also tested the interaction between LDL-C and RC groups. Analyses were conducted with SAS version 9.4 (SAS Institute). A *P*-value <0.05 (2-tailed) was considered significant.

## 3 Results

### 3.1 Baseline characteristics

The baseline characteristics of the participants are shown in Tables 1, 2. Among the 13,383 participants aged 20 years or older, the mean age was 45.72 years, and 52% of participants were female. Compared with participants in the lowest quartile of RC, those with higher levels of RC were older, less educated, and more likely to be male, White, and Hispanic. They were more likely to be current smokers, have physical activity levels, and were at greater risk than baseline patients for hypertension and diabetes. From the perspective of high-risk cardiovascular risk factors, participants with higher levels of RC were prone to have lower HDL-C levels, and higher levels of TC, TG, LDL-C, blood pressure, fasting glucose, glycated hemoglobin, and BMI (Table 2).

**Table 1:** Baseline demographic and lifestyle characteristics of the study population according to the RC level.

Characteristics	RC level, mg/dl								*P* value
	Q1, <14.26 mg/dl		Q2, 14.26–19.77 mg/dl		Q3, 19.77–29.80 mg/dl		Q4, ≥29.80 mg/dl		
	No. (%)	SE	No. (%)	SE	No. (%)	SE	No. (%)	SE	
No. of participants	3,345		3,346		3,346		3,346		
Age, mean, year	38.0	0.40	40.8	0.50	45.5	0.60	48.1	0.50	<0.001
Sex (*n*, %)									<0.001
Male	1,407 (39.7)	1.20	1,504 (46.6)	1.70	1,616 (51.6)	1.20	1,855 (59.7)	1.20	
Female	1,938 (60.3)	1.20	1,842 (53.4)	1.70	1,730 (48.4)	1.20	1,491 (40.3)	1.20	
Race/ethnicity (*n*, %)									<0.001
Non-Hispanic White	1,108 (71.5)	2.10	1,251 (76.2)	1.50	1,333 (75.6)	1.60	1,458 (79.8)	1.40	
Non-Hispanic Black	1,442 (16.7)	1.20	1,020 (11.0)	0.80	802 (9.4)	0.50	549 (6.0)	0.50	
Mexican-American	650 (3.8)	0.30	933 (5.2)	0.50	1,059 (5.9)	0.50	1,215 (6.7)	0.60	
Others	145 (8.1)	1.40	142 (7.5)	0.90	152 (9.1)	1.40	124 (7.6)	1.10	
Educational level (*n*, %)									<0.001
Less than high school	526 (7.4)	0.70	728 (9.0)	0.70	834 (11.9)	1.00	976 (13.3)	1.00	
High school	1,614 (43.3)	1.60	1,575 (45.8)	1.60	1,606 (49.7)	1.60	1,555 (49.3)	1.60	
College or higher	1,205 (49.2)	1.70	1,043 (45.2)	1.70	906 (38.5)	1.60	815 (37.4)	1.70	
Ratio of family income to poverty (*n*, %)									<0.001
≤1.30	965 (16.4)	1.40	1,015 (16.4)	1.20	967 (17.0)	1.30	1,008 (17.5)	1.30	
1.31–3.50	1,356 (40.9)	1.70	1,339 (42.4)	1.60	1,362 (43.5)	1.40	1,353 (44.2)	1.50	
>3.50	735 (36.3)	2.00	680 (35.2)	1.70	706 (33.8)	1.70	661 (31.5)	2.10	
Unknown	289 (6.4)	0.70	312 (6.0)	0.70	311 (5.7)	0.70	324 (6.7)	0.60	
Marital status (*n*, %)									<0.001
Married	1,805 (60.6)	1.7	2,000 (64.1)	1.50	2,085 (66.5)	1.30	2,330 (71.9)	1.60	
Widowed	226 (3.8)	0.40	267 (5.5)	0.60	340 (6.9)	0.50	355 (7.5)	0.70	
Divorced	280 (8.8)	0.70	241 (7.9)	0.70	274 (8.3)	0.80	242 (8.1)	0.87	
Single	1,024 (26.5)	1.60	830 (22.5)	1.40	643 (18.1)	1.10	414 (12.5)	1.15	
Unknown	10 (0.2)	0.10	8 (0.1)	0.10	4 (0.1)	0.00	5 (0.1)	0.04	
Smoking status (*n*, %)									<0.001
Never	1,903 (53.2)	1.30	1,707 (49.4)	1.50	1,606 (42.4)	1.50	1,473 (39.8)	1.40	
Former	572 (18.9)	0.90	694 (21.8)	1.20	835 (25.8)	1.70	955 (30.6)	1.20	
Active	870 (27.8)	1.40	945 (28.8)	1.30	905 (31.7)	1.30	918 (29.6)	1.30	
Alcohol intake (*n*, %)									<0.001
None	2,418 (66.1)	1.50	2,543 (73.2)	1.90	2,533 (72.8)	2.00	2,625 (77.2)	1.40	
Moderate	289 (9.8)	1.10	279 (8.7)	0.90	290 (8.9)	1.00	250 (8.1)	0.70	
Heavy	521 (20.9)	1.10	429 (15.7)	1.20	382 (14.3)	1.10	357 (11.2)	0.90	
Missing	117 (3.2)	0.80	95 (2.4)	0.50	141 (4.1)	1.00	114 (3.6)	0.60	
Physical activity (*n*, %)									<0.001
Inactive	594 (12.1)	1.20	692 (14.1)	1.20	689 (13.5)	0.90	767 (15.3)	1.10	
Insufficient	1,392 (44.6)	1.40	1,409 (43.5)	1.70	1,494 (47.3)	1.20	1,455 (46.9)	1.80	
Sufficient	1,359 (43.3)	1.50	1,245 (42.4)	1.70	1,163 (39.2)	1.40	1,124 (37.8)	1.80	
HEI-2010	62.60	0.40	63.20	0.40	63.00	0.40	64.60	0.50	<0.001
Total energy intake (TEI), Kcal	2,156.4	47	2,193.8	29	2,125.3	34	2,141.7	28	<0.001
BMI categories (*n*, %)									<0.001
<25.0 kg/m^2^	1,978 (67.7)	1.40	1,585 (53.5)	1.20	1,052 (36.5)	1.50	668 (21.4)	1.40	
25.0–30 kg/m^2^	897 (23.5)	1.30	1,044 (29.6)	1.40	1,295 (36.2)	1.10	1,398 (42.3)	1.40	
≥30 kg/m^2^	462 (8.8)	0.80	713 (16.9)	0.90	988 (27.2)	1.20	1,272 (36.1)	1.40	
Diabetes (*n*, %)									<0.001
No	3,092 (94.5)	0.60	2,947 (91.6)	0.70	2,697 (85.5)	1.10	2,387 (78.3)	1.40	
Yes	253 (5.5)	0.60	399 (8.4)	0.70	649 (14.5)	1.10	959 (21.7)	1.40	
Hypertension (*n*, %)									<0.001
No	2,796 (90.0)	0.60	2,610 (83.9)	1.00	2,322 (76.1)	1.20	2,048 (65.5)	1.50	
Yes	549 (10.0)	0.60	736 (16.1)	1.00	1,024 (23.9)	1.20	1,298 (34.5)	1.50	
Hyperlipidemia (*n*, %)									<0.001
No	3,140 (94.1)	0.60	2,883 (85.6)	0.90	1,938 (57.4)	1.50	4 (0.1)	0.10	
Yes	205 (5.9)	0.60	463 (14.4)	0.90	1,408 (42.6)	1.50	3,342 (99.9)	0.10	
Cholesterol-lowering drugs, (*n*, %)									<0.001
No	3,318 (99.3)	0.20	3,296 (98.3)	0.30	3,255 (97.3)	0.40	3,199 (95.6)	0.50	
Yes	27 (0.7)	0.20	50 (1.7)	0.30	91 (2.7)	0.40	147 (4.4)	0.50	

Values are means (SE) for continuous variables or percentages (SE) for categorical variables and are weighted except No. of participants. HEI, healthy eating index; TEI, total energy intake; BMI, body mass index.

**Table 2:** Distribution of cardiovascular risk factors of the study population according to the RC level.

Risk factors	RC level, mg/dl								*P* value
	Q1, <14.26 mg/dl		Q2, 14.26–19.77 mg/dl		Q3, 19.77–29.80 mg/dl		Q4, ≥29.80 mg/dl		
	No. (%)	SE	No. (%)	SE	No. (%)	SE	No. (%)	SE	
Systolic blood pressure, mmHg	115.71	0.43	119.29	0.48	123.26	0.58	127.98	0.56	<0.001
Diastolic blood pressure, mmHg	71.48	0.28	73.10	0.27	75.06	0.35	77.90	0.26	<0.001
Fasting glucose, mg/dl	91.01	0.43	94.33	0.81	98.76	0.63	107.35	1.16	<0.001
Glycated hemoglobin, %	5.10	0.02	5.21	0.03	5.39	0.03	5.64	0.04	<0.001
CRP (*n*, %)									<0.001
<1.00 mg/L	3,109 (95.2)	0.60	3,034 (93.5)	0.50	2,953 (90.0)	0.80	3,004 (91.3)	0.70	
≥1.00 mg/L	213 (4.2)	0.50	272 (5.2)	0.40	364 (8.9)	0.80	311 (8.0)	0.70	
Lipid profiles									
TC, mg/dl	183.24	1.1	191.51	1.08	206.93	1.00	229.13	1.35	<0.001
HDL-C, mg/dl	59.78	0.58	53.03	0.40	47.87	0.39	41.14	0.39	<0.001
TG, mg/dl	60.12	0.35	92.61	0.29	134.77	0.47	260.36	2.42	<0.001
LDL-C, mg/dl	111.58	1.51	123.01	1.45	135.27	1.02	141.09	1.84	<0.001
RC, mg/dl	11.52	0.06	16.84	0.04	24.16	0.08	46.81	0.43	<0.001

Values are means (SE) for continuous variables or percentages (SE) for categorical variables and are weighted except No. of participants. CRP, C-reactive protein; TC, total cholesterol; HDL-C, high-density lipoprotein cholesterol; TG, triglyceride; LDL-C, low-density lipoprotein cholesterol; RC, remnant cholesterol.

### 3.2 Association of RC level with all-cause and cardiovascular mortality

During a median follow-up period of 26.6 years (maximum follow-up 31 years), 5,044 participants died, of which 1,741 died of CVD (1,409 of IHD and 332 of stroke) and 1,126 of cancer. In the crude model, elevated RC was associated with a higher risk of all-cause mortality and cause-specific mortality in a graded manner (Table 3). However, such association was attenuated when we considered covariates. In a fully adjusted model, compared to participants with the lowest quartile of RC (<14.26 mg/dl), the hazard of all-cause mortality was significant for those with RC of 19.77–29.80 mg/dl [HR 1.21 (95% CI: 1.07–1.37)] and RC ≥29.80 mg/dl [HR 1.23 (95% CI: 1.07–1.42)]. The highest risk for CVD mortality was observed in participants with RC ≥29.80 mg/dl [HR 1.22 (95% CI: 0.97–1.53)].

**Table 3:** Association between RC levels with all-cause, CVD, IHD, stroke and cancer mortality among 13,383 individuals.

RC levels, mg/dl				
Outcomes	Q1, <14.26 mg/dl, *n* = 3,345	Q2, 14.26–19.77 mg/dl, *n* = 3,346	Q3, 19.77–29.80 mg/dl, *n* = 3,346	Q4, ≥29.80 mg/dl, *n* = 3,346
All-cause mortality				
Deaths/person years	846/38,591	1,102/56,357	1,434/80,897	1,662/157,147
Model 1	1 (ref.)	1.47 (1.25, 1.73)	2.26 (2.02, 2.53)	2.76 (2.38, 3.20)
Model 2	1 (ref.)	1.14 (0.98, 1.33)	1.30 (1.15, 1.47)	1.38 (1.20, 1.58)
Model 3	1 (ref.)	1.14 (0.98, 1.32)	1.28 (1.14, 1.44)	1.36 (1.18, 1.56)
Model 4	1 (ref.)	1.10 (0.95, 1.26)	1.21 (1.07, 1.37)	1.23 (1.07, 1.42)
CVD mortality				
Deaths/person years	308/38,591	366/56,357	477/80,897	590/157,147
Model 1	1 (ref.)	1.39 (1.10, 1.76)	2.36 (1.89, 2.94)	3.20 (2.52, 4.06)
Model 2	1 (ref.)	1.03 (0.79, 1.34)	1.26 (0.97, 1.64)	1.51 (1.18, 1.92)
Model 3	1 (ref.)	1.02 (0.78, 1.32)	1.24 (0.97, 1.58)	1.46 (1.14, 1.87)
Model 4	1 (ref.)	0.94 (0.74, 1.20)	1.10 (0.87, 1.40)	1.22 (0.97, 1.53)
IHD mortality				
Deaths/person years	245/38,591	305/56,357	382/80,897	477/157,147
Model 1	1 (ref.)	1.50 (1.11, 2.04)	2.58 (2.00, 3.32)	3.44 (2.66, 4.43)
Model 2	1 (ref.)	1.11 (0.81, 1.53)	1.39 (1.05, 1.83)	1.62 (1.25, 2.09)
Model 3	1 (ref.)	1.10 (0.81, 1.51)	1.36 (1.05, 1.77)	1.57 (1.21, 2.05)
Model 4	1 (ref.)	1.03 (0.77, 1.36)	1.22 (0.95, 1.57)	1.32 (1.03, 1.69)
Stroke mortality				
Deaths/person years	63/38,590	61/56,357	95/80,897	113/157,147
Model 1	1 (ref.)	0.996 (0.64, 1.55)	1.59 (0.97, 2.61)	2.39 (1.45, 3.95)
Model 2	1 (ref.)	0.73 (0.45, 1.19)	0.84 (0.48, 1.47)	1.12 (0.66, 1.88)
Model 3	1 (ref.)	0.71 (0.45, 1.14)	0.81 (0.47, 1.38)	1.08 (0.65, 1.81)
Model 4	1 (ref.)	0.66 (0.42, 1.05)	0.71 (0.43, 1.18)	0.89 (0.55, 1.43)
Cancer mortality				
Deaths/person years	199/38,591	233/56,357	338/80,897	356/157,147
Model 1	1 (ref.)	1.27 (0.93, 1.74)	2.32 (1.85, 2.92)	2.24 (1.68, 2.98)
Model 2	1 (ref.)	1.04 (0.77, 1.41)	1.47 (1.16, 1.87)	1.23 (0.95, 1.60)
Model 3	1 (ref.)	1.05 (0.78, 1.40)	1.44 (1.12, 1.87)	1.23 (0.95, 1.60)
Model 4	1 (ref.)	1.03 (0.77, 1.39)	1.40 (1.08, 1.82)	1.17 (0.90, 1.52)

Values are *n* or hazard ratio (95% confidence interval) and are weighted except No. of deaths/person-years. Model 1: Crude model. Model 2: adjusted for age, sex, and race/ethnicity. Model 3: model 2 + education, family income level, TEI, HEI, smoking status, alcohol intake, and physical activity. Model 4: model 3+ BMI, diabetes, and hypertension. TEI, total energy intake; HEI, Healthy Eating Index, BMI, body mass index.

We further assessed the association of RC levels with IHD and stroke mortality (Table 3). Compared to the lowest quartile of RC level, the highest quartile of RC (≥29.80 mg/dl) was closely associated with the highest IHD mortality with HR of 1.32 (95% CI: 1.03–1.69). However, we did not observe a significant association between RC and stroke mortality. For cancer mortality, compared with RC level <14.26 mg/dl, those with RC levels of 19.77–29.80 mg/dl had a higher risk of cancer mortality [HR 1.40 (95% CI: 1.08–1.82)].

**Figure 1:**
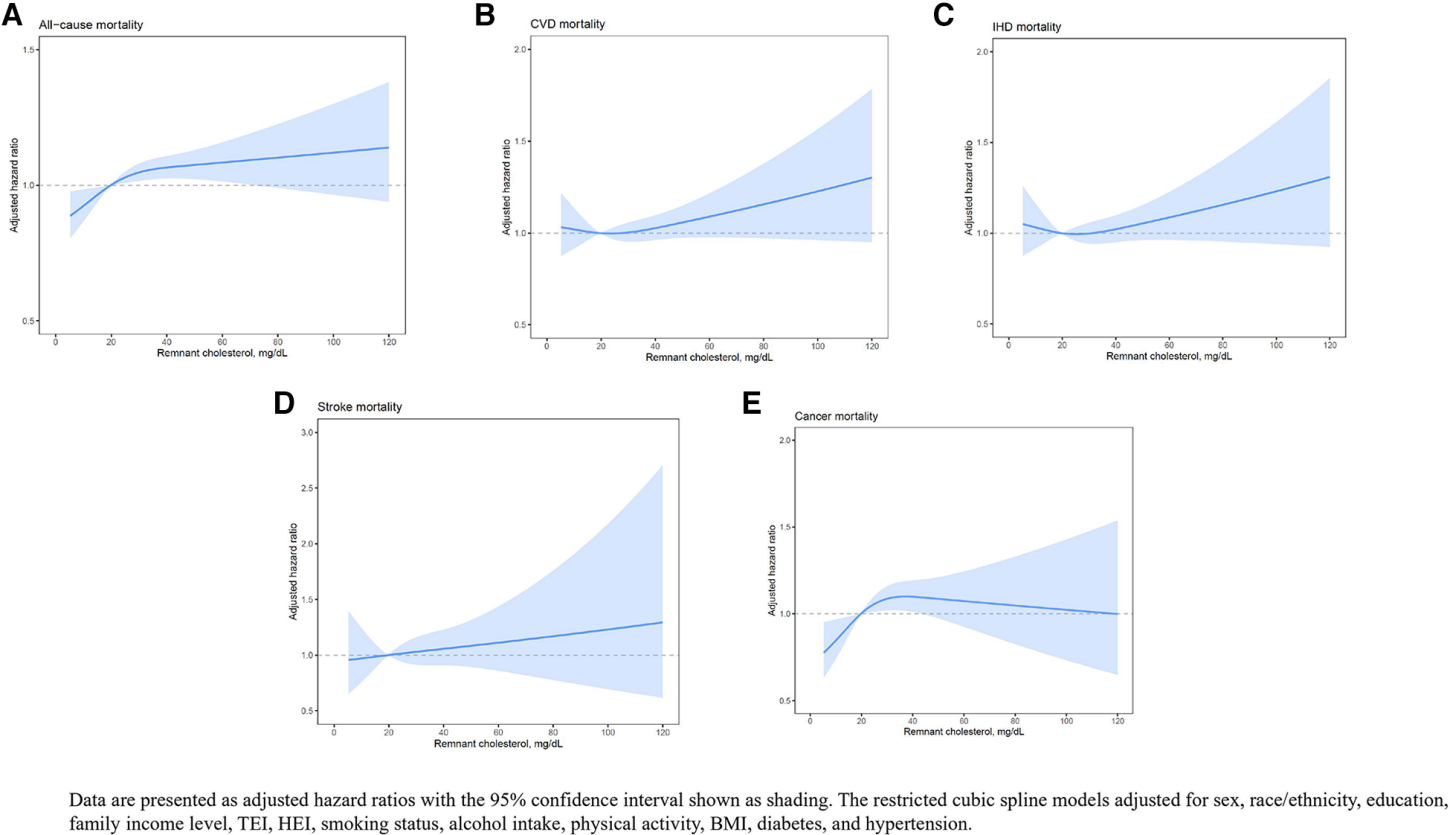
The restricted cubic spline model which presents the nonlinear association of RC with all-cause and cause-specific mortality.

We also plotted smooth splines using a restricted cubic spline model to present the nonlinear association of RC with all-cause and cause-specific mortality (Figure 1).

### 3.3 Secondary analysis

Analysis stratified by sex, age, race/ethnicity, BMI, smoking status, alcohol consumption, physical activity level, diabetes, and hypertension further demonstrates the associations of RC with all-cause, CVD, and IHD mortality (Supplementary Tables S1–S3). Female participants within 60 years old, with obesity, less active, individuals without hypertension and diabetes, and ever-smokers or ever-drinkers had a higher risk for CVD and IHD mortality. In addition, the interaction between RC and age (*P* for interaction <0.0001) has a significant impact on the mortality of CVD. Compared with people over 60 years old, the association between RC and mortality of CVD in young people was stronger.

In addition, this study conducted sensitivity analyses after adjusting LDL-C, ApoB and excluding participants who died within four years of follow-up (Supplementary Table S4–S6) and found that the results remained robust.

### 3.4 Interaction analysis between RC and LDL-C on mortality

The lipid guidelines recommend cut-off LDL-C value of 130 mg/dl (3.4 mmol/L) for the general population. Based on the RC quartiles, we observed that the risk of CVD mortality tends to increase after the RC level is at 19.77 mg/dl. Therefore, we defined the RC threshold as 20 mg/dl. As shown in Table 4, participants with higher LDL-C and higher RC levels had a high risk of CVD and IHD mortality (HR 1.30; 95% CI: 1.04–1.61 and HR 1.38; 95% CI: 1.09–1.73, respectively) compared with the reference group (low LDL-C and low RC). Among those with low LDL-C but high RC levels, the risk of CVD and IHD mortality was HR 1.24 (95% CI: 1.03–1.50) and HR 1.28 (95% CI: 1.01–1.61), respectively, compared to the reference group.

**Table 4:** Risk of mortality based on categories of LDL-C and remnant-C levels.

Outcomes	LDL-C ≤130 mg/dl and RC ≤20 mg/dl *n* = 5,766	LDL-C ≤130 mg/dl and RC >20 mg/dl *n* = 5,094	LDL-C >130 mg/dl and RC ≤20 mg/dl *n* = 1,045	LDL-C >130 mg/dl and RC >20 mg/dl *n* = 1,478
All-cause mortality				
HR (95% CI)	1 (ref.)	1.18 (1.05, 1.31)	0.94 (0.83, 1.07)	1.08 (0.92, 1.26)
*p*-int	1 (ref.)	0.0044	0.33	0.36
CVD mortality				
HR (95% CI)	1 (ref.)	1.24 (1.03, 1.50)	1.07 (0.85, 1.33)	1.30 (1.04, 1.61)
*p*-int	1 (ref.)	0.028	0.58	0.022
IHD mortality				
HR (95% CI)	1 (ref.)	1.28 (1.01, 1.61)	1.07 (0.82, 1.40)	1.38 (1.09, 1.73)
*p*-int	1 (ref.)	0.039	0.59	0.0081
Stroke mortality				
HR (95% CI)	1 (ref.)	1.09 (0.77, 1.56)	1.00 (0.55, 1.81)	0.95 (0.57, 1.59)
*p*-int	1 (ref.)	0.61	0.999	0.84
Cancer mortality				
HR (95% CI)	1 (ref.)	1.16 (0.96, 1.39)	0.81 (0.59, 1.12)	1.33 (1.01, 1.75)
*p*-int	1 (ref.)	0.12	0.20	<0.001

To assess the risk of mortality by low and high categories of LDL-C and RC, HRs were plotted relative to the lowest risk category (LDL-C≤130 mg/dl and RC≤20 mg/dl). Data were adjusted for age, sex, race/ethnicity, education, family income level, TEI, HEI, smoking status, alcohol intake, physical activity, BMI, diabetes, and hypertension. TEI, total energy intake; HEI, Healthy Eating Index. *p*-int, *p* for interaction(Interaction between RC and LDL-C).

## 4 Discussion

In this large nationally representative cohort study, we found that high levels of RC were associated with an increased risk of all-cause, CVD, and IHD mortality with a median follow-up of 26.6 years (maximum 31 years). Elevated RC levels were associated with an increased risk of CVD and IHD mortality regardless of whether LDL-C levels were in the normal range. Our research findings suggested the clinical importance of detecting the residual cardiovascular risk relation with RC and considering LDL-C-related risk as we open a new era of targeted lipid-lowering treatments.

Several studies have shown that elevated RC levels are a risk factor for CVD and all-cause mortality (6, 7, 23–27). The literature found that for patients with IHD, when RC ≥1.5 mmol/L, the risk of all-cause mortality increased by 50% compared to the normal RC group (25). The evidence from 90,000 Danish general participants suggested that non-fasting RC better predicts all-cause mortality than LDL-C (27). The PREDIMED study indicated that the level of RC increase by every 0.26 mmol/L, then the risk of major CVD in the obese and diabetic population increase by 21%, and it is independent of LDL-C (26). These studies would suggest that RC is a factor in residual cardiovascular risk in addition to LDL-C (28). The Chinese Kailuan study indicated that the risk of all-cause mortality increased from 1.10 to 1.23 times for RC in the second to fourth quartile, from 0.94 to 1.22 times for CVD, and from 1.03 to 1.32 times for IHD (29). Notably, the risk of CVD due to elevated RC levels was slightly lower in this paper compared to the Chinese Kailuan study, which may be related to the younger mean age of the participants and the lower prevalence of cardiovascular risk factors (e.g., diabetes and hypertension) in this study. However, similar findings were not found when the stroke was used as the outcome event. In addition, ischemic stroke has multiple causes, 15%–40% of which are caused by atherosclerosis, which is present in 90% of myocardial infarctions. Therefore, the association of RC with ischemic/hemorrhage cerebrovascular morbidity and mortality needs to be further explored in future prospective studies.

Our findings showed an increased risk of cancer mortality when the RC was in the range of 19.77–29.8 mg/L after adjusting for confounding factors. A Danish study found that elevated TG levels increase the risk of cancer mortality (30). However, other studies suggested that elevated RC levels were related with increased CVD mortality but not cancer mortality (31, 32). Notably, the relationship between RC and cancer risk has yet to be extensively studied.

Mechanistically, TG levels and RC levels are highly correlated, but TG is quickly metabolized *in vivo*, whereas cholesterol is not. Therefore, researchers believe that it is RC, not TG *per se*, that increases CVD risk (8, 33). Different from LDL-C, RC is rich in cholesterol due to its structure, such as relatively large volume and high quantity, and does not need to be modified by oxidation, preferentially entering the arterial intima and depositing in the intima, leading to increased cholesterol levels promoting atherogenesis (34). However, unfortunately, the mechanisms by which elevated RC levels lead to CVD risk and all-cause mortality are still not fully elucidated. According to advances in recent decades, several hypotheses have been proposed. First, atherosclerosis begins with damage to the intima and inflammatory response. High levels of RC in plasma increase the permeability of the intima. After transendothelial cells accumulate in the intima of arteries and are taken up by macrophages, accelerating the formation of foam cells (35), which become part of the atherosclerotic plaque. Second, RC promotes the progression of atherosclerosis by inducing an inflammatory response (36). Finally, RC also activates the coagulation cascade (11), which promotes platelet aggregation and microthrombus formation (37). These processes may lead to an increased risk of CVD and all-cause mortality.

The main strength of this study is the nationally representative design, which may favor extrapolating our results to the general population in the US. Observations over 30 years, the present study was able to identify and quantify long-term risks related to RC levels. We considered various confounding factors and performed sensitivity analyses. Notably, according to data from NHANES III, the prevalence of lipid-lowering treatments in the US is only 3.4%, which hardly affects the RC level of adults (38). Therefore, the correlation between RC and outcomes is less influenced by confounding factors such as baseline lipid-lowering therapies. Several limitations should be acknowledged. First, this study was observational, and causal inference regarding RC levels and the risk of death should be cautious and lipid profile was only assessed once at baseline, thus precluding the observations of RC changes with mortality risk. Second, due to NHANES not providing information on participants of stroke subtypes (ischemic or hemorrhagic), this may limit the interpretation of RC levels and stroke subtype deaths. Third, considering ENHANES adopts a complex and diverse design, it is not possible to consider weights, parameters, repeated sampling, etc. to conduct competing risk analysis in this mode. Finally, the effect of residual confounders from unmeasured factors could not be entirely excluded because of the observational nature.

## 5 Conclusion

In summary, in this large nationally representative cohort study of 13,383 US adults with up to a maximum follow-up of 31 years, elevated blood RC has relevance to increased mortality from all-causes, CVD, and IHD. Such association was independent of socioeconomic factors, lifestyles, and history of diseases, and remained robust across the LDL-C stratum that requires greater public health attention. Measuring RC levels might favor clinical assessment of early CVD risk. With new lipid-lowering drugs such as statins, preprotein convertase chymotrypsin 9 inhibitors, apolipoprotein antisense oligodeoxynucleic acid, and microsomal triglyceride transporter protein inhibitors, new interventions are available to reduce RC levels. To this end, further studies in other countries or ethnic populations are warranted to evaluate whether lifestyle modification or medical treatment for RC will promote cardiovascular health, improve the overall quality of life, and curb the subsequent risk of death. Our results highlight the negative impact of high RC levels on cardiovascular outcomes, shedding new insight on the importance of lipid control in both clinical and lifestyle settings. Further studies are warranted to investigate the optimal range of RC levels for cardiovascular health in the general population. Such investigations may provide critical information for developing more targeted interventions and guidelines to improve cardiovascular health outcomes.

## Data Availability

The datasets presented in this study can be found in online repositories. The names of the repository/repositories and accession number(s) can be found in the article/Supplementary Material.
