# Supplementary material for: Long-term association of remnant cholesterol with all-cause and cardiovascular disease mortality: a nationally representative cohort study
Source: Front Cardiovasc Med. 2024 Jul 8;11:1286091. doi: 10.3389/fcvm.2024.1286091 (PMC11284489; doi:10.3389/fcvm.2024.1286091)
Supplement: Supplementary file 1 [file Datasheet1.pdf]

Supplementary Material

Supplementary Table 1. Stratified analyses of the associations of RC with all-cause mortality among 13,383 individuals.

Supplementary Table 2. Stratified analyses of the associations of RC with CVD mortality among 13,383 individuals.

Supplementary Table 3. Stratified analyses of the associations of RC with IHD mortality among 13,383 individuals.

Supplementary Table 4. Sensitivity analyses of the association between RC and mortality excluding those who developed died within four years of follow-up (n=12,855).

Supplementary Figure 1. Flow chart of participants in this study.

Supplementary Table 1. Stratified analyses of the associations of RC with all-cause mortality

	RC levels, mg/dL				
**All-cause mortality**	**Q1, <14.26 mg/dL, HR (95% CI)**	**Q2, 14.26-19.77 mg/dL, HR (95% CI)**	**Q3, 19.77-29.80 mg/dL, HR (95% CI)**	**Q4, ≥29.80 mg/dL, HR (95% CI)**	***P* value for interaction**
**Age, years**					
<60	1.00 (ref.)	1.20 (0.96, 1.50)	1.44 (1.20, 1.73)	1.55 (1.28, 1.88)	<0.0001
≥60	1.00 (ref.)	1.10 (0.90, 1.35)	1.20 (0.98, 1.46)	1.06 (0.91, 1.24)	
**Sex**					
Men	1.00 (ref.)	1.20 (0.96, 1.50)	1.19 (1.01, 1.39)	1.24 (1.00, 1.54)	0.54
Women	1.00 (ref.)	1.00 (0.86, 1.17)	1.24 (1.01, 1.52)	1.21 (0.98, 1.50)	
**Race/ethnity**					
White	1.00 (ref.)	1.18 (0.96, 1.44)	1.36 (1.18, 1.57)	1.27 (1.06, 1.52)	0.44
Non-White	1.00 (ref.)	1.08 (0.94, 1.25)	1.25 (1.04, 1.49)	1.26 (1.08, 1.47)	
**Obesity**					
BMI <30 kg/m^2	1.00 (ref.)	1.07 (0.81, 1.42)	1.17 (0.88, 1.55)	1.02 (0.78, 1.35)	0.17
BMI ≥30 kg/m^2	1.00 (ref.)	1.07 (0.76, 1.51)	1.28 (0.94, 1.75)	1.35 (0.98, 1.85)	
**Smoking status**					
Never smoker	1.00 (ref.)	1.18 (0.99, 1.40)	1.24 (1.05, 1.48)	1.08 (0.89, 1.32)	0.17
Ever smoker	1.00 (ref.)	1.17 (0.94, 1.45)	1.48 (1.27, 1.74)	1.46 (1.20, 1.78)	
**Alcohol consumption**					
Never drinker	1.00 (ref.)	1.14 (0.99, 1.31)	1.36 (1.22, 1.51)	1.22 (1.06, 1.41)	0.98
Ever drinker	1.00 (ref.)	1.34 (0.93, 1.94)	1.42 (1.07, 1.88)	1.67 (1.20, 2.32)	
**Physical activity level**					
Lower	1.00 (ref.)	1.32 (1.10, 1.59)	1.30 (1.13, 1.49)	1.34 (1.13, 1.60)	0.94
Meets or exceeds guidelines	1.00 (ref.)	1.01 (0.81, 1.25)	1.43 (1.19, 1.71)	1.27 (1.03, 1.58)	
**Diabetes**					
Yes	1.00 (ref.)	1.05 (0.75, 1.49)	1.25 (0.91, 1.73)	1.33 (0.99, 1.78)	0.31
No	1.00 (ref.)	1.21 (1.02, 1.44)	1.39 (1.24, 1.57)	1.33 (1.14, 1.55)	
**Hypertension**					
Yes	1.00 (ref.)	0.96 (0.81, 1.15)	1.13 (0.94, 1.36)	0.98 (0.81, 1.17)	0.0001
No	1.00 (ref.)	1.25 (0.98, 1.59)	1.44 (1.24, 1.68)	1.48 (1.22, 1.78)	

HR, hazard ratio; CI, confidence interval. Adjusted for age, sex, race/ethnicity, education, family income level, TEI, HEI, smoking status, alcohol intake, physical activity, BMI, diabetes, and hypertension.

Abbreviations: HEI, healthy eating index; TEI, total energy intake; BMI, body mass index.

Supplementary Table 2. Stratified analyses of the associations of RC with CVD mortality

RC levels, mg/dL					
**CVD**	**Q1, <14.26 mg/dL HR (95% CI)**	**Q2, 14.26-19.77 mg/dL, HR (95% CI)**	**Q3, 19.77-29.80 mg/dL, HR (95% CI)**	**Q4, ≥29.80 mg/dL, HR (95% CI)**	***P* value for interaction**
**Age, years**					
<60	1.00 (ref.)	1.10 (0.73, 1.66)	1.49 (0.98, 2.27)	1.91 (1.21, 3.03)	<0.0001
≥60	1.00 (ref.)	0.93 (0.71, 1.21)	1.01 (0.79, 1.29)	0.91 (0.74, 1.12)	
**Sex**					
Men	1.00 (ref.)	0.95 (0.66, 1.37)	1.03 (0.73, 1.46)	1.16 (0.78, 1.73)	0.93
Women	1.00 (ref.)	0.92 (0.67, 1.26)	1.14 (0.79, 1.63)	1.19 (0.90, 1.58)	
**Race/ethnity**					
White	1.00 (ref.)	1.01 (0.76, 1.35)	1.30 (0.99, 1.71)	1.24 (0.91, 1.69)	0.58
Non-White	1.00 (ref.)	1.02 (0.77, 1.35)	1.02 (0.63, 1.65)	1.19 (0.87, 1.63)	
**Obesity**					
BMI <30 kg/m^2	1.00 (ref.)	1.01 (0.77, 1.34)	1.19 (0.91, 1.56)	1.13 (0.86, 1.48)	0.28
BMI ≥30 kg/m^2	1.00 (ref.)	0.92 (0.57, 1.50)	1.28 (0.84, 1.95)	1.37 (0.91, 2.06)	
**Smoking status**					
Never smoker	1.00 (ref.)	0.996 (0.76, 1.31)	1.07 (0.77, 1.49)	0.90 (0.65, 1.25)	0.026
Ever smoker	1.00 (ref.)	1.05 (0.76, 1.45)	1.44 (1.07, 1.93)	1.56 (1.11, 2.17)	
**Alcohol consumption**					
Never drinker	1.00 (ref.)	1.14 (0.99, 1.31)	1.36 (1.22, 1.51)	1.22 (1.06, 1.41)	0.035
Ever drinker	1.00 (ref.)	1.34 (0.93, 1.94)	1.42 (1.07, 1.88)	1.67 (1.20, 2.32)	
**Physical activity level**					
Lower	1.00 (ref.)	1.23 (0.88, 1.72)	1.32 (1.003, 1.73)	1.41 (1.01, 1.98)	0.54
Meets or exceeds guidelines	1.00 (ref.)	0.83 (0.62, 1.11)	1.14 (0.75, 1.72)	1.07 (0.75, 1.52)	
**Diabetes**					
Yes	1.00 (ref.)	1.12 (0.58, 2.17)	1.09 (0.59, 2.01)	1.42 (0.74, 2.72)	0.62
No	1.00 (ref.)	1.03 (0.79, 1.34)	1.36 (1.06, 1.74)	1.27 (1.01, 1.58)	
**Hypertension**					
Yes	1.00 (ref.)	0.96 (0.81, 1.15)	1.13 (0.94, 1.36)	0.98 (0.81, 1.17)	0.019
No	1.00 (ref.)	1.25 (0.98, 1.59)	1.44 (1.24, 1.68)	1.48 (1.22, 1.78)	

HR, hazard ratio; CI, confidence interval. Adjusted for age, sex, race/ethnicity, education, family income level, TEI, HEI, smoking status, alcohol intake, physical activity, BMI, diabetes, and hypertension.

Abbreviations: HEI, healthy eating index; TEI, total energy intake; BMI, body mass index.

Supplementary Table 3. Stratified analyses of the associations of RC with IHD mortality

IHD	RC levels, mg/dL				
	Q1, <14.26 mg/dL HR (95% CI)	Q2, 14.26-19.77 mg/dL, HR (95% CI)	Q3, 19.77-29.80 mg/dL, HR (95% CI)	Q4, ≥29.80 mg/dL, HR (95% CI)	*P* value for interaction
**Age, years**					
<60	1.00 (ref.)	1.17 (0.74, 1.86)	1.68 (1.05, 2.69)	2.16 (1.33, 3.51)	<.0001
≥60	1.00 (ref.)	1.02 (0.75, 1.41)	1.11 (0.85, 1.46)	0.97 (0.78, 1.21)	
**Sex**					
Men	1.00 (ref.)	0.91 (0.61, 1.36)	1.07 (0.75, 1.53)	1.14 (0.75, 1.75)	0.61
Women	1.00 (ref.)	1.13 (0.78, 1.63)	1.38 (0.91, 2.10)	1.44 (1.01, 2.05)	
**Race/ethnity**					
White	1.00 (ref.)	1.16 (0.83, 1.62)	1.51 (1.12, 2.03)	1.41 (1.001, 1.98)	0.25
Nonwhite	1.00 (ref.)	0.97 (0.72, 1.31)	1.03 (0.61, 1.75)	1.17 (0.82, 1.66)	
**Obesity**					
BMI <30 kg/m^2	1.00 (ref.)	1.15 (0.84, 1.57)	1.32 (0.98, 1.78)	1.26 (0.96, 1.67)	0.71
BMI ≥30 kg/m^2	1.00 (ref.)	0.80 (0.44, 1.45)	1.19 (0.77, 1.83)	1.20 (0.74, 1.93)	
**Smoking status**					
Never smoker	1.00 (ref.)	1.10 (0.77, 1.56)	1.11 (0.74, 1.65)	0.91 (0.64, 1.30)	0.023
Ever smoker	1.00 (ref.)	1.14 (0.78, 1.67)	1.68 (1.22, 2.30)	1.74 (1.19, 2.55)	
**Alcohol consumption**					
Never drinker	1.00 (ref.)	0.98 (0.76, 1.26)	1.14 (0.86, 1.51)	1.10 (0.84, 1.45)	0.003
Ever drinker	1.00 (ref.)	1.24 (0.62, 2.48)	1.70 (0.91, 3.19)	2.30 (1.23, 4.32)	
**Physical activity level**					
Lower	1.00 (ref.)	1.32 (0.89, 1.95)	1.43 (1.04, 1.96)	1.58 (1.10, 2.26)	0.39
Meets or exceeds guidelines	1.00 (ref.)	0.92 (0.65, 1.31)	1.28 (0.78, 2.10)	1.07 (0.70, 1.64)	
**Diabetes**					
Yes	1.00 (ref.)	1.15 (0.57, 2.32)	1.17 (0.59, 2.32)	1.55 (0.74, 3.25)	0.902
No	1.00 (ref.)	1.13 (0.84, 1.51)	1.51 (1.18, 1.93)	1.34 (1.05, 1.71)	
**Hypertension**					
Yes	1.00 (ref.)	0.94 (0.73, 1.20)	1.12 (0.88, 1.43)	1.08 (0.86, 1.37)	0.0013
No	1.00 (ref.)	1.07 (0.73, 1.57)	1.31 (0.92, 1.87)	1.36 (0.93, 1.99)	

HR, hazard ratio; CI, confidence interval. Adjusted for age, sex, race/ethnicity, education, family income level, TEI, HEI, smoking status, alcohol intake, physical activity, BMI, diabetes, and hypertension.

Abbreviations: HEI, healthy eating index; TEI, total energy intake; BMI, body mass index.

Supplementary Table 4. Sensitivity analyses of the association between RC and mortality excluding those who developed died within four years of follow-up (n=12,855)

	RC levels, mg/dL			
**Outcomes**	**<14.22 mg/dL, n=3213**	**14.22-19.72 mg/dL, n=3214**	**19.72-29.76 mg/dL, n=3214**	**≥29.76 mg/dL, n=3214**
**All-cause mortality**	1 (ref.)	1.12 (0.98, 1.29)	1.21 (1.05, 1.39)	1.26 (1.08, 1.47)
**CVD mortality**	1 (ref.)	1.01 (0.78, 1.32)	1.13 (0.87, 1.48)	1.30 (1.004, 1.69)
**IHD mortality**	1 (ref.)	1.07 (0.77, 1.49)	1.21 (0.91, 1.62)	1.37 (1.03, 1.82)
**Stroke mortality**	1 (ref.)	0.77 (0.47, 1.25)	0.82 (0.47, 1.41)	1.04 (0.60, 1.78)
**Cancer mortality**	1 (ref.)	0.99 (0.74, 1.33)	1.31 (1.000, 1.71)	1.17 (0.90, 1.53)

Data were presented as hazard ratios (95% CIs) with adjustment of age, sex, race/ethnicity, education, family income level, TEI, HEI, smoking status, alcohol intake, physical activity, BMI, diabetes, and hypertension.

Abbreviations: HEI, healthy eating index; TEI, total energy intake; BMI, body mass index; CVD, cardiovascular disease; IHD, ischemic heart disease.

Supplementary Figure 1. Flow chart of participants in this study.

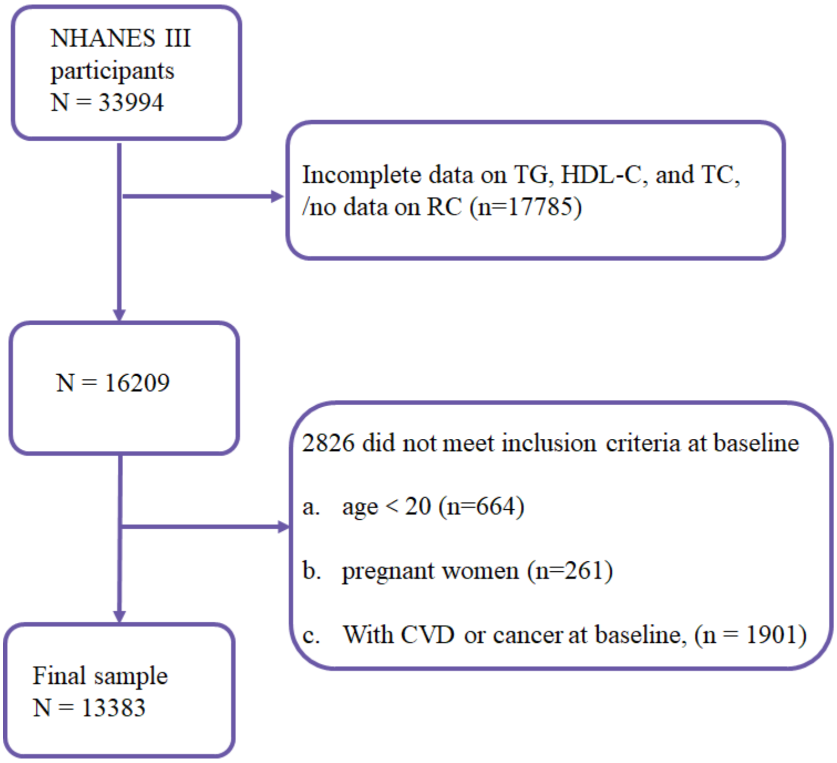

Abbreviations: CVD, cardiovascular disease; HDL-C, high-density lipoprotein-cholesterol; LDL-C, low-density lipoprotein-cholesterol; NHANES, National Health and Nutrition Examination Survey; TC, total cholesterol; TG, triglyceride; RC, remnant cholesterol.
